# 3D ultrasound as a surgical quality control of conization in patients with severe dysplasia: a prospective study

**DOI:** 10.1007/s00404-020-05718-9

**Published:** 2020-08-01

**Authors:** Anna M. Dückelmann, Julia Wordell, Rolf Richter, Jalid Sehouli

**Affiliations:** 1 Department of Gynecology, Medical University of Berlin, Augustenburger Platz 1, 13353 Berlin, Germany; 2 Department of Gynecology, Ruppiner Kliniken, Fehrbelliner Str. 38, 16816 Neuruppin, Germany

**Keywords:** 3D ultrasound, Cervical dysplasia, Conization, Cervical volume, Cervical length, Transvaginal ultrasound

## Abstract

**Purpose:**

To compare the techniques for cone measurement with ultrasound to determine the size of the resected tissue and to evaluate parameters which may be relevant for stratifying women at risk who need surveillance when pregnant.

**Methods:**

The present study included women with a pathological cervical biopsy. Cervical length and volume were determined by transvaginal ultrasound prior to conization. The pathologist measured the volume of the removed tissue by the fluid displacement technique and using a ruler. A repeat transvaginal ultrasound was performed during a follow-up visit. Factors affecting cone volume as well as the correlation between measurement techniques were analyzed.

**Results:**

A total of 28 patients underwent cervical excision treatment. The mean cervical volumes measured sonographically before and after the operation were 17.72 ± 7.34 and 13.21 ± 5.43 cm^3^, respectively. The proportion of volume excised was 25.50 ± 17.43%. A significant correlation was found between the cone depth and the cone volume measured by the fluid displacement technique, and histopathologically and sonographically measured difference in cervical volume. The interobserver reliability coefficient was > 0.9. Analyzing influential parameters, only age affected the extent of cone volume and the correlation between the three measurement techniques.

**Conclusion:**

Commonly applied techniques of cervical and cone measurement are equivalent and interchangeable. Our ultrasound data show variety in the volume and length of the cervix, and in the proportion of the volume excised at conization. Ultrasound measurements may help the surgeon to estimate not only the dimension of the remaining cervix but also its function.

## Introduction

The implementation of cervical carcinoma screening in various countries and the introduction of HPV vaccination have contributed to a significant decrease in the incidence and mortality rates for this cancer [1]. Nevertheless, cervical cancer remains a threat to women’s lives globally [2].

A decrease in invasive cervical cancer is ensured by the timely diagnosis and appropriate treatment of high-grade preinvasive cervical lesions using cytology. The methods of choice for treating cervical intraepithelial neoplasia (CIN) are loop electrosurgical excision procedure (LEEP) and carbon dioxide laser cervical excision [3]. These procedures allow the degree of abnormality in the excised tissue to be thoroughly evaluated, as well as the excision margins, while preserving healthy tissue. In addition, these techniques are inexpensive and can be performed as outpatient procedures under local anesthesia. Cure rates up to 95% are achieved [4].

Excisional treatment methods may lead to a higher risk of adverse obstetric outcomes in affected fertile women [5–8]. Women who have undergone cervical excision treatment have relatively short mid-trimester cervical lengths [9] and are at an increased risk of preterm delivery [10]. The goal of the operative procedure must be to guarantee oncological safety while minimizing the morbidity and individual risk of preterm birth. Parameters affecting the cervical function are not yet clarified. The cervical length, volume, or proportion of excised tissue may be relevant for stratifying women at risk who need surveillance when pregnant. Different measurement techniques are applied worldwide but the possible exchange between those techniques has not yet been investigated. While performing the conization, the surgeon is not able to determine, even when measuring the excised cone, how much of the cervix he/she removed. The extent of cervical damage, however, appears to be more accurately reflected by measuring the proportions of the volume excised than the absolute cone volume [11]. The dimensions of the excised specimen are important predictors of subsequent premature delivery [5, 12].

After cervical damage, a regenerative/healing process has been described to originate from epithelial and stromal tissue repair cells [13], and is complete by the sixth post-operative month [14]. The efficacy of this process is important, as good structural and anatomical recovery may reduce the risk of negative obstetric outcomes.

The aim of this prospective study was, first, to compare the techniques for cone measurement with the determination of resected tissue size by ultrasound, and to evaluate the length and volume of the cervix before and after conization for CIN. As a further step, we wanted to show that by means of 3D ultrasound, the remaining cervix can be additionally evaluated in proportion to the cone, under consideration of the regeneration process.

## Materials and methods

This study included a total of 38 women with abnormal Pap smear results who were referred to a large Cervical Dysplasia Center. Written informed consent was obtained from all patients. The approval of the ethics committee of the Charité hospital Campus Mitte in Berlin was obtained in advance of the planned study. High-grade CIN was diagnosed at our institution after colposcopy-directed cervical punch biopsy using Münchener nomenclature III [15]. The background characteristics of each patient recorded at inclusion were: age, parity, tobacco use, oral contraceptive use, HPV status, history of CIN treated by conization, preferences for subsequent pregnancy, and post-operative complications requiring additional surgery.

Cervical length and volume were determined by transvaginal ultrasound (TVUS) prior to conization. We evaluated differences in the cervix size (length/volume) prior to treatment between the participating individuals. The volume of the removed tissue was measured by the fluid displacement technique before fixation in formalin. Briefly, the parts of the cone were submerged in a fluid-filled tube, and we recorded the difference in fluid levels [16]. The pathologist then measured the removed tissue using a ruler [17]. All magnification-assisted cervical excisional treatments were performed under strict colposcopic guidance [18] with rounded loops (1.5–2.5 cm) chosen based on the type of transformation zone and the tissue area to be removed. Histopathological diagnosis was established, and we recorded the highest dysplasia grade and lesion margins.

An ultrasound follow-up date has already been arranged at the time of surgery, patients missing this appointment were repeatedly reminded by phone. The cytological and colposcopic control visit was performed independently of the sonographic examination. During the follow-up visit, a repeat TVUS by the sonographer blinded to the preconization measurements was obtained using the same protocol as before treatment to assess the volume of the cervix post-treatment and the proportion of the volume excised: cone volume (= volume before excision − volume after excision)/volume cervix before excision. The cone length was calculated as the ultrasound measurement at follow-up subtracted from the ultrasound measurement before conization.

**Fig. 1:**
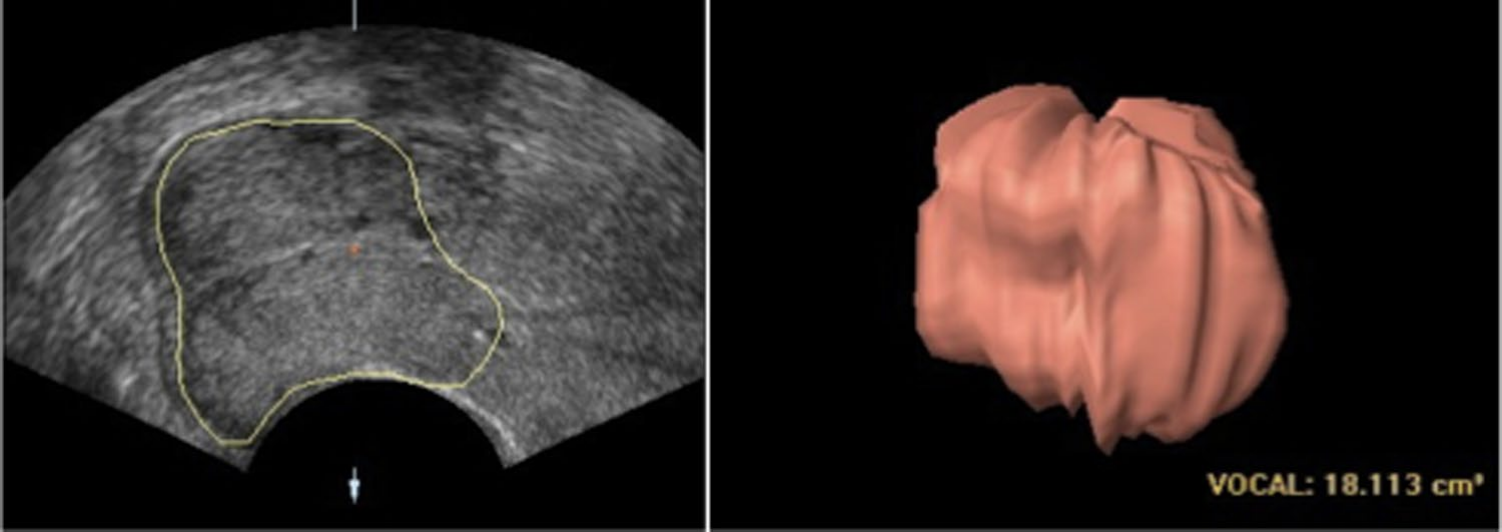
Cervix measurement. **a** Midsagittal view of the cervix after conization (B-mode image). Cervical contour is outlined. **b** Calculation of cervical volume by Virtual Organ Computer-Aided Analysis (VOCAL).

The ultrasound imaging was performed by one examiner (A.D.), who has more than 5 years of experience in gynecological ultrasound, using a 4–9-MHz 3D transvaginal probe and a Voluson 730 Expert ultrasound system (GE Healthcare, Zipf, Austria). In 15 cases, a second examiner evaluated cases to analyze inter-observer reliability. For the exam, the women were in the dorsal lithotomy position with an empty urinary bladder [16]. The probe was slowly introduced into the vagina to obtain a satisfactory image without exerting pressure on the cervix. We measured the length of the cervix in the sagittal plane as the straight distance between the internal and external os. Cervical volume was measured using a three-dimensional and automated technique, virtual organ computer-aided analysis (VOCAL; 3D View, version 2.0, GE Healthcare, Milwaukee, WI), which was preferred over the cylinder geometric formula for volume determination because of its comparable accuracy [19] and the fact that only one investigator (A.D.) examined all cases [20]. VOCAL is a software application in which semiautomatic volume calculations are performed via fixed axis rotation through a number of sequential steps [21]. In the multiplanar view of the cervix, the coronal image was manipulated to demonstrate the true mid-sagittal view, including the entire endocervical canal with the internal and external os. The upper limit of the cervix was defined arbitrarily as the plane perpendicular to the cervical canal at the inferior limit of the endometrial line. We used fast volume acquisition to minimize artifacts caused by patient movement. The volume of the cervix was determined after its contour was traced on six images of the uterus obtained by 30° rotations [22] (see Fig. 1). The optimally acquired volumes were saved for later analysis offline by a surgeon blinded to the results.

At follow-up, regeneration of the tissue deficit at the site of the cervical crater was defined according to Papoutsis [16]: % Regeneration = [(*a* − *b*)/*a*] × 100, where *a* is the cervical deficit immediately after LEEP and *b* is the tissue deficit at follow-up. The initial tissue deficit is represented by the size of the cone, whereas the tissue deficit at follow-up is the difference in cervical volume measured at follow-up and baseline (before conization). The sonographer performing the follow-up TVUS assessments was blinded to the preconization measurements. The regeneration process was investigated by dividing the initial study population into two groups: time interval between the first and second ultrasound examination ≤ 4 weeks and ≥ 15 weeks. The influence of regeneration on the different methods of cone measurement was assessed by box plots.

Analyses were performed using statistical software SPSS V.25 (SPSS, IBM, Chicago, Illinois, USA, 2018). The inter-operator and intra-operator agreement for ultrasound measurements were evaluated using the Wilcoxon signed ranks test, Kendall’s tau-b, Spearman’s correlation coefficient (CC), and the variation coefficient. An intraclass CC and interclass CC > 0.75 was considered a reliable consensus [23]. Spearman’s CC was used to compare continuous non-normally distributed variables. Significance was set at *P* < 0.05.

Factors that affect the reduction in dimensions of the cervix and the different methods of calculating cone volume were identified using the Mann–Whitney *U* test.

## Results

This prospective, observational study was conducted at a University Hospital. We included 38 patients who underwent cervical excision treatment at our institution; 10 were lost to follow-up. Therefore, this study included 28 women who completed follow-up (Table 1).

**Table 1:** Demographics and clinical characteristics

Characteristic	Patients
Age	32.93 ± 9.17
20–25	5 (17.86%)
> 25–30	10 (35.71%)
> 30–35	4 (14.29%)
> 35–40	3 (10.71%)
> 40	6 (21.43%)
Parity	
0	13 (56.52%)
≥ 1	10 (43.48%)
Smoking	
Yes	13 (68.42%)
No	6 (31.58%)
Preoperative cervical findings	
CIN 2	5 (20.83%)
CIN 3	19 (79.17%)
Previous conization	2 (7.14%)
HPV preoperative	
Yes	25 (89.29%)
No	3 (10.71%)
Oral contraceptive	
Yes	10 (58.82%)
No	7 (41.18%)
Child wish	
Yes	6 (33.33%)
No	12 (66.66%)
Final histology of the cone specimen	
CIN 1	4 (14.29%)
CIN 2	9 (32.14%)
CIN 3	15 (53.57%)
Positive endocervical margine	1 (6.67%)
Interval between surgery and follow-up (weeks)	23.54 ± 20.07
Cytology at follow-up	
Normal	27 (96.43%)
Pap IVa	1 (3.57%)

Data are presented as *n* (%) or mean ± SD.

In all cases, the indication for surgery was a pathological biopsy (CIN II/III). In all but one case, the cytological and colposcopic control 3 months after the procedure was unremarkable. The exceptional case underwent a second conization because of not completely removed CIN III without any further problems.

The cervical characteristics obtained by sonography at baseline (before cervical excision treatment) and follow-up are reported in Table 2. The mean time interval between surgery and follow-up was 24 weeks (Table 1). We found a significant correlation between the length of the cervix measured by 2D TVUS and volume of the cervix measured by VOCAL both preoperatively and postoperatively (Table 3).

**Table 2:** Sonographic cervical characteristics at baseline and follow-up

	Mean ± SD	Min	Median	Max
Baseline cervical length (cm)	3.1018 ± 0.69167	1.6	3.07	4.62
Baseline cervical volumen (cm^3^)	17.71557 ± 7.341257	2.73	16.62	38.45
Cervical length at follow-up (cm)	2.5796 ± 0.49880	1.58	2.615	3.5
Cervical volumen at follow-up (cm^3^)	13.2064 ± 5.43200	2.76	13.115	23.95
Excised volumen measured sonographically (%)	25.4992 ± 17.42845	0.87	21.06	57.7
Cone length (mm)	7.43 ± 4.442	2	7	22
Cone volumen measured histopathologically (cm^3^)	2.2148 ± 2.26634	0.22	1.61	10.55
Cone volume according to Archimedes (ml)	1.0022 ± 0.72715	0.2	0.8	2.7

**Table 3:** Spearman-Rho correlation coefficient

	Cervical length	Cervical volume
Pre-LEEP measurement	0.975	0.939
Post-LEEP measurement	0.987	0.932
Difference	0.856	0.782

The mean ± SD cone volume was 1.0022 ± 0.72715 ml by the fluid displacement technique, 2.2148 ± 2.26634 cm^3^ according to histopathological measurements, and 4.5091 ± 4565.01704 cm^3^ when the difference in cervical volume was measured sonographically before and after the surgical procedure. Around one fourth of the cervical volume was removed on average (Table 2). We found no correlation between the cone depth and the histopathological diagnosis (CC = −0.10). However, a significant correlation was found between the cone depth and the cone volume measured by the displacement method, the histopathologically measured volume, and the sonographically measured difference in cervical volume (Table 4). We also found a significant correlation between the volume measured by the displacement technique and the volume measured histopathologically (CC = 0.775). We did not find a correlation between the volume measured by the displacement method and the difference in cervical length (CC = 0.069) or the difference in cervical volume (CC = − 0.111).

**Table 4:** Influential parameters

Cone volume	Measured histopathologically		Measured sonographically		Measured according to Archimedes	
Depth of cone	CC 0.791	*p *= 0.000	CC 0.483	*p* = 0.009	CC 0.592	*p* = 0.003
Age	CC 0.49	*p* = 0.008	CC 0.129	*p* = 0.511	CC 0.105	*p* = 0.633
Histopathological diagnosis	CC 0.208	*p* = 0.288	CC − 0.346	*p* = 0.071	CC 0.378	*p* = 0.075
Parity	CC 0.181	*p* = 0.409	CC − 0.212	*p* = 0.331	CC 0.166	*p* = 0.459

Assessing the intra- and inter-observer variability in the ultrasound measurements, we found a high degree of reliability. The intra-rater reliability (*n* = 9) was very good, with a high correlation (tau-*b* = 0.93), and a low variation coefficient between 0.01 and 0.104. The inter-observer reliability (*n* = 15) was also very good, with a CC of 0.975 and a low variation coefficient between 0 and 0.11 for preoperative evaluation of the cervical length, and a CC of 0.987 and variation coefficient between 0 and 0.13 for post-operative evaluation of the cervical length.

The reliability of 3D measurement of the volume of the cervix before and after conization demonstrated clinical relevance. Concerning the pre- and post-operative measurement of cervical volume by 3D ultrasound, the correlation was very good (CC = 0.939, variation coefficient 0.02–0.19 and CC = 0.932, variation coefficient 0.02–0.45, respectively). The correlation was also good regarding the difference in cervical volume measured sonographically (CC = 0.782, variation coefficient − 0.94–1.21, see Table 3).

**Fig. 2:**
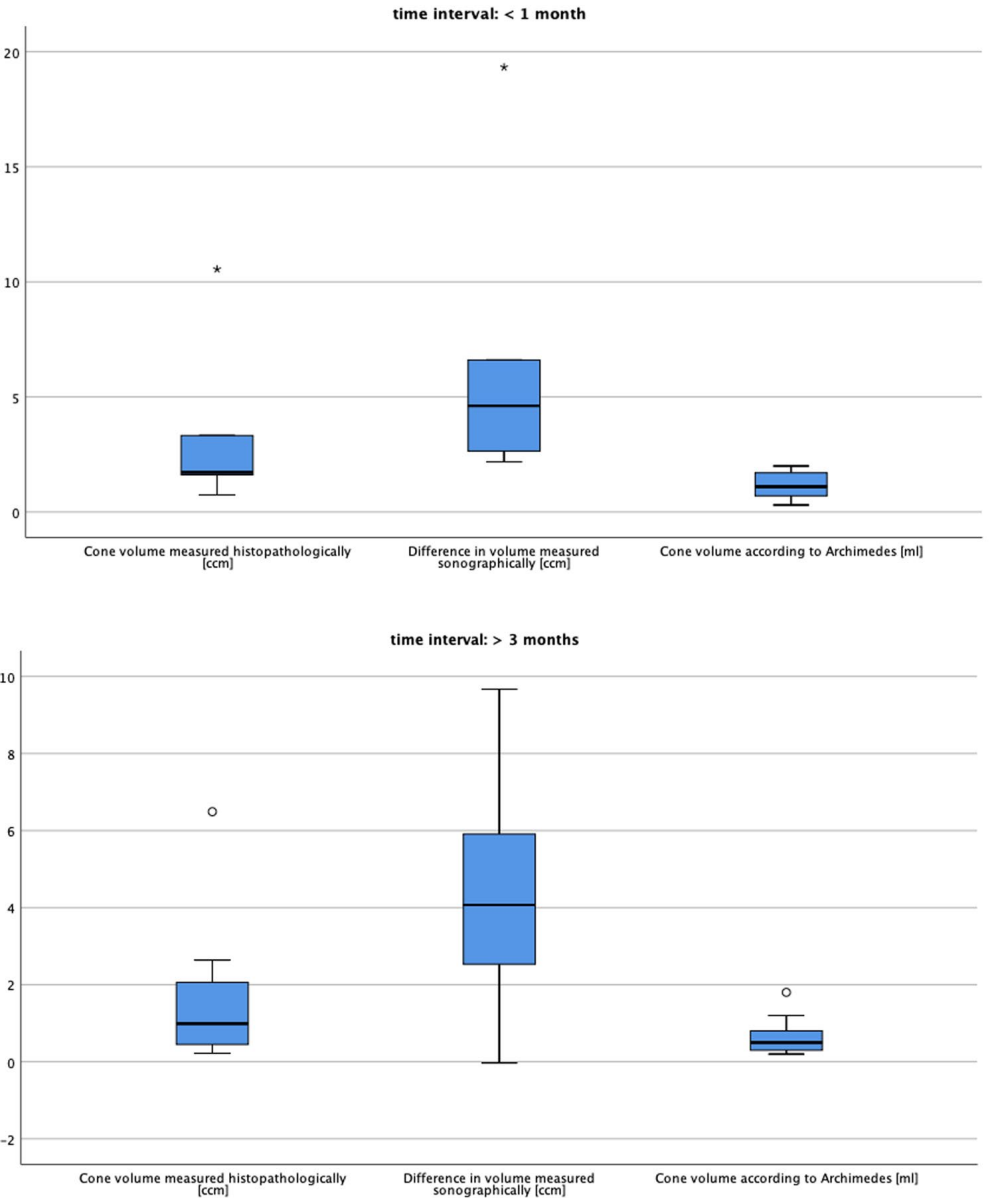
**a**, **b** Effect of tissue regeneration.

In consideration of the time interval between surgery and follow-up, we analyzed the effect of regeneration. The study group was divided into two groups: group A in which the time between surgery and the follow-up examination was ≤ 4 weeks (*n* = 7), and group B in which the time interval was ≥ 15 weeks (*n* = 15). The correlation between the difference in cervical volume measured sonographically and the cone volume was higher in group A (CC = 0.700) than in group B (CC = − 0.039), in which the regeneration process already had an effect (Fig. 2a, b). Due to the small sample size, we could not analyze the extent of regeneration according to time and its influential factors.

**Fig. 3:**
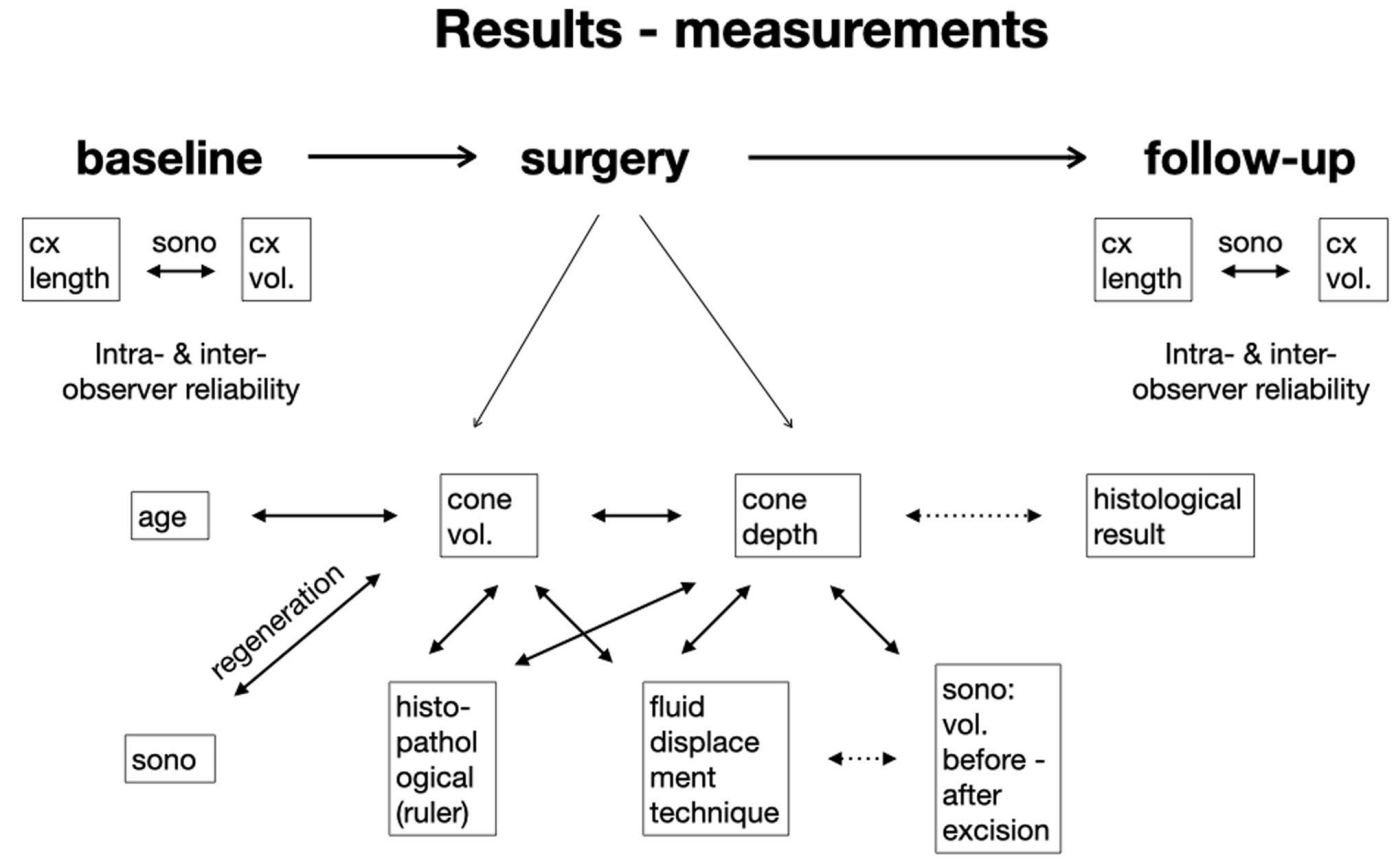
Measurements at baseline, time of surgery and follow-up. *vol* Volume, *cx* cervical, *sono* ultrasound, *double-headed arrow* correlation, *dotted double-headed arrow* no correlation

Analyzing the different factors, we only found a significant effect of age on the extent of cone volume and on the correlation between the three measurement techniques (Fig. 3). We did not find a correlation between age and the pathological diagnosis (CC = 0.216). No influence of parity, histopathological diagnosis, HPV, smoking, or the use of oral contraceptives was shown on the proportion of volume excised.

## Discussion

We compared different techniques of cervical measurement before and after conization for the first time and were able to show the reliability of ultrasound measurements. Our ultrasound data show great variety in the volume and length of the cervix and in the proportion of the volume excised at conization, which seems to more accurately reflect the extent of damage to the cervix [11] and to be more relevant for the post-operative function of the cervix than the cone volume itself. Few studies have reported the effect of LEEP on cervical volume. Carcopino et al. [17] showed that the smaller uterine and cervical dimensions correlate with the volume of tissue removed by conization. The cervical volume is an index of the functional reserve of the cervix, making changes clinically important [24, 25].

Cervical excision is associated with an increased risk of prematurity and perinatal morbidity in future pregnancies, with a direct correlation between the amount of tissue excised and the risk of preterm birth [6, 7, 9, 10, 12, 26]. The resection height is important in the development of functional cervical insufficiency after [27]. A 2006 meta-analysis [6] noted a significant increase in the risk of preterm delivery with an excision depth > 10 mm. A Danish registry study of 3605 pregnancies after LLETZ demonstrated an approximate 6% increase in the risk of preterm delivery per additional millimeter of excised tissue [28]. With a mean thickness of 7.43 ± 4.442 mm for the excised cone in our cohort, deep excisions were rare. Only four patients had a LEEP specimen exceeding 10 mm in thickness.

Interestingly, the extent of the excised volume tissue in our study depended on the age of the patient only; thus, the surgeons operated differently depending on age and not based on objective criteria, such as histopathological diagnosis, transformation zone, and the size of the lesion.

In contrast to the study by Ahmed et al. [20], we found a correlation between the length of the cervix measured by 2D transvaginal ultrasound and the volume of the cervix measured by VOCAL. Changes in the length or diameter of the cervix may affect the volume measurements in different proportions, possibly because the irregular shape of the cervix has an effect.

The proportion of the cervical volume excised influences not only cervical composition and function [29], but also cervical regeneration. Using MRI, Founta et al. [30] reported an association between the deficit in the regenerated cervix and the proportional volume excised/ablated. Papoutsis et al. [16] used 3D transvaginal sonography to estimate cervical regeneration, demonstrating that the deficit 6 months after treatment correlates with the proportion of cervical volume excised. They reported a 1.37% reduction for each 1% increase in excised cervical volume. To achieve > 75% regeneration of the tissue in the cervical crater at 6 months, the excised volume must not exceed 14% of the initial volume of the cervix. Nicolas et al. [31] evaluated cervical regeneration 1 month and 6 months after the procedure and demonstrated a cervical “re-growth” process, with 71% mean regeneration at 6 months. In our study, we indirectly demonstrated the effect of regeneration via the difference in cervical volume measured sonographically and the cone volume as a factor of time. Taken together, the results suggest that the healing process following LEEP significantly affects the cervix.

We showed that the commonly applied techniques of cervical measurement are actually equivalent and interchangeable. Ultrasound measurements as a recommended standard of procedure before and after conization have been easily implemented at our institution and may help the surgeon to evaluate/estimate not only the dimension of the remaining cervix but also its function.

The main limitation of our study is the small sample size, which did not allow us to perform specific subgroup analyses, such as the influence of the extent of the volume excised on regeneration. We could not quantify the cervical tissue removed during coagulation for hemostasis; even if we used coagulation moderately, and only in the case of a single bleeding vessel, this would be a limitation. To limit bias, we did not include any subjective tissue loss assessed by the colposcopist in the total volume of the cone. The main technical challenge with 3D volume postprocessing was indistinct demarcation lines, especially those between the upper cervix and the lower uterine segment [20]. The resulting inclusion of non-cervical tissue, such as the lower uterine segment or vaginal wall, inside the lines may have had the consequence of inaccurate volumes and explain the relative overestimation of cervix volume by VOCAL. To reduce such subjectivity, we arbitrarily defined the upper limit of the cervix as the plane perpendicular to the cervical canal at the inferior limit of the endometrial line.

Finally, our study did not consider pregnancy outcomes, particularly the risk of prematurity, as only 6 women in our cohort became pregnant during the observation period.

## Outlook

Cervical dimensions could easily be measured by ultrasound and cone dimensions measured in common practice, and could act as quality control for the surgeon. Therefore, ultrasound could improve both surgical technique and long-term outcomes. The clinician can calculate the percentage of cervix that was removed by conization based on a single 3D ultrasound of the cervix before conization and the cervical cone volume determined after excision. It is crucial that cervical excision treatments be performed under colposcopic guidance to reduce the amount of healthy tissue removed while still achieving clinical efficacy. This can be achieved by taking into consideration the type of transformation zone and desire for future pregnancies.

A standardized surveillance protocol following LEEP is not yet available for pregnant women, and the only currently recognized risk factor is cone length, which does not address individual variability in cervix length or volume and the proportion of removed tissue, or the structural changes in the regenerated tissue. Comprehensive studies are necessary to establish a limit for the amount of excised tissue in relation to obstetric morbidity, as well as define treatment success in terms of recurrent CIN or cancer. Only then will it be possible to establish appropriate counseling for women undergoing cervical excision to treat high-grade CIN in regards to the risk of prematurity in a subsequent pregnancy and to decrease patient morbidity.

## Data Availability

All data and material are available on request.
